# Economic Feasibility of Mixed-Species Grazing to Improve Rangeland Productivity

**DOI:** 10.3390/ani11051226

**Published:** 2021-04-23

**Authors:** Kayla Hintze, Courtney Bir, Derrell Peel

**Affiliations:** Department of Agricultural Economics, Oklahoma State University, Stillwater, OK 74074, USA; kayla.hintze@okstate.edu (K.H.); derrell.peel@okstate.edu (D.P.)

**Keywords:** multi-species grazing, beef cattle, meat goat, cost-benefit analysis

## Abstract

**Simple Summary:**

The encroachment of woody plants, including the eastern redcedar, in the central Great Plains is reaching critical levels. This encroachment impacts the profitability of cattle grazing operations, and potentially the ability to meet consumer demand for beef products due to lower stocking rates. Even though chemical and fire control are currently being used, the incorporation of small ruminants such as goats can help control the spread of woody plants, while providing an additional source of revenue and protein. In this analysis, we take a data driven approach to analyzing the potential for success utilizing different combination of chemicals, fire, breeding goats and stocker goats to control woody plant encroachment. Given our assumptions, the combination with the highest net present value was cattle, controlled burning and breeding goats.

**Abstract:**

Pasture and grazing land in the southern and central Great Plains is being invaded by woody species, especially eastern redcedar. As a result of woody plant encroachment, cattle production on native rangeland is becoming less profitable because stocking rates must be decreased. Eastern redcedar encroachment can be controlled by grazing management, herbicide use, prescribed fire, mechanical control and mixed species grazing. This study utilizes traditional management practices, prescribed fire and three types of mixed species grazing operations to determine the most economically feasible way to manage redcedar encroachment on rangeland. The cost-benefit analysis in this study found that the source of redcedar management on rangeland with the highest net present value was the use of a breeding goat operation in which goats were grazed alongside cattle with the use of prescribed fire. This suggests that producers who are fighting redcedar encroachment will likely be able to implement a mixed species grazing operation with breeding goats to better manage their land and increase returns.

## 1. Introduction

The United States is the global leader in beef production, making it a vital part of the U.S. economy [1]. As the demand rises for beef, producers must search for ways to produce more beef on the same amount, or less land while at least maintaining economic returns. However, pasture and grazing land is being invaded by woody species such as eastern redcedar (*Juniperus virginiana*). As a result of woody plant encroachment, rangeland is becoming less profitable because stocking rates must be decreased [2]. Redcedar trees prevent cattle forage growth by shading over grass and decreasing water availability which would otherwise be used by forage species [3]. Economic losses from reduced forage production in 2013 were estimated to be 205,000,000 dollars [3]. Woody plant cover in the southern and central Great Plains has increased by nearly 1.5% per year over the past century [4]. Originally, the grasslands of the Great Plains were home to bison, elk, pronghorn and deer [5]. These large grazing herbivores provided a sustainable mix of grazers, browsers and mixed feeders [5]. Additionally, wildfires were intense and frequent, also contributing to woody plant control [5]. Changes in land management as homesteading became prevalent, in addition to the removal of native herbivores has allowed for increasing encroachment of woody plants [5]. When cattle, which are grazers, are the only animal on the grassland, the roles of browser and mixed feeder are left empty [5].

There are a variety of ways to control eastern redcedar encroachment, but the effectiveness of the proposed methods has not been extensively studied. Grazing management, herbicides, prescribed fires and complementary forage systems are a few of the possible ways to mitigate eastern redcedar encroachment [6]. Prescribed fires in conjunction with grazing livestock is pyric herbivory [7]. This method changes the grazing patterns of livestock species [7]. For example, immediately after burning, the area burned is not ideal for grazing, but as grass sprouts livestock will return to grazing the previously burned areas [7]. Each of these control methods has potential issues. A study performed to identify how herbicides and fire could be used to control woody plant encroachment found that the use of herbicides was not sufficient to prevent the spread of woody plants [8]. The herbicide Tebuthiuron is used to control woody plant encroachment, but the use of this herbicide was shown to be ineffective [8]. Even though herbicides have been used for many years as a form of woody plant control, in general, this method is expensive, time consuming and not always effective [8]. The use of prescribed fires for woody plant control has been popular in the past and will continue as a popular choice because it is often less expensive than mechanical control, and potentially more effective [6]. However, both prescribed fires and manual control are labor intensive [9]. Adequate fuel levels are required to kill larger eastern redcedar trees. Grazed rangeland generally lacks adequate fuel levels to kill large cedar trees [10]. With the fuel load in Oklahoma, prescribed fires are only capable of killing trees under about 5 feet tall [10].

Another proposed method to control eastern redcedar encroachment is through the introduction of an animal species that consumes woody plants. Mixed species grazing may offer one solution to controlling woody plants while grazing cattle. Mixed species grazing is a type of grazing system utilizing two species of animals to increase the productivity of land. Mixed species grazing does not exhaust the land because each animal is able to utilize different plants more efficiently. An experiment by McMahan [11] studied the difference in forage type consumption of cattle, goats, sheep and deer. McMahan [11] found that throughout all four seasons, sheep, goats and deer consumed significantly more browse than cattle did. Browse is defined as food from woody perennials [12]. The diet of a sheep appeared to be more similar to the diet of cattle, whereas goats and deer consumed primarily browse [11]. In situations where significant woody species are present, grazing goats with cattle may increase rangeland carrying capacity by 70% because goats prefer brushy forage whereas cattle are more likely to consume grasses [13].

A mixed species grazing operation can be composed of any combination of at least two species of animals; this analysis evaluates the combination cattle and goats. Consumption of goat meat has increased throughout the world because it has unique nutritional values when compared to other red meats [14]. Chevon (goat meat) has lower fat and cholesterol contents, making it more appealing to health-conscious consumers. In the United States, an influx of immigrants from goat consuming countries has increased the demand for goat. In many cases, producers in the United States can receive premium for Halal goats, as Muslim communities in the US grow [15]. Another species often used to graze with cattle is sheep. Goats were chosen to graze alongside cattle for this study because of their increasing popularity and greater dietary difference from cattle. Goats are browsers as opposed to grazers such as sheep, meaning they prefer to consume leaves and woody plants and therefore compete less with cattle for grass. Not only will the goats contribute to woody plant control, but they can be sold for additional revenue at the end of each production year, increasing potential returns. According to Coffey [16], one or two mature goats can be added per head of cattle without reducing cattle stocking rates, due to differences in grazing habits.

Mixed species grazing can offer more economic stability and greater returns as opposed to managing a single species herd [17]. The objective of this study is to determine if mixed species grazing can be an economically profitable way to prevent eastern redcedar encroachment in the Great Plains and improve rangeland productivity. Five types of operations are studied, all of which incorporate a cow-calf operation. The first two operations or production enterprise mixes (hereby technologies) are traditional management, and the use of prescribed fire. The last three technologies incorporate goats alongside the cattle and prescribed fire.

## 2. Materials and Methods

In this case, 5 technologies are evaluated in this study to determine which method of woody plant encroachment results in the highest expected economic returns. The technologies included are: (1) the standard woody plant control method with cattle grazing, (2) use of prescribed fire with cattle grazing, (3) use of fire, stocker goats and cattle grazing with goat feed supplementation during the entire season, (4) use of fire, stocker goats and cattle grazing with goat feed supplementation in the late season only and (5) the use of fire, breeding goats and cattle grazing with goats only supplemented when necessary for breeding. The assumptions made for each technology are summarized in Table 1. The use of fire and any goat operation combined with cattle would incur the greatest costs over technology 1. However, the benefit of each method is evaluated as the woody plant population decreases, consequently improving the land. Furthermore, there is an additional revenue opportunity associated with selling the goats used in the mixed species grazing scenario. Technology 1 (control group) for this study is a cattle grazing operation using standard eastern redcedar control methods such as mechanical control and herbicide use [6]. This study is based on an experiment currently in progress at Oklahoma State University studying rangeland improvement. Several sets of data were obtained from these experiments, but outside data sources were used to account for data which has not been collected yet.

The cattle-only operation will be considered the base, and each additional technology beyond that point will incur extra costs and potentially additional revenue. It is assumed that the test pasture is 180 acres, which was chosen based on the study conducted at Oklahoma State University [26]. Forage production varies greatly across individual pastures and should be evaluated for each scenario. For the purpose of this study, it is assumed that cattle require 11 acres per head, resulting in 16 cows on the 180-acre pasture [18]. According to the Meat Goat Production Handbook [24], 0.5 to 2 breeding goats can be added per head of cattle without decreasing cattle stocking rate. Because stocker goats are being utilized to consume woody plants, 4 stocker goats will be added per head of cattle in technologies 3 and 4. Stocker goats are smaller and will only be grazed on the pasture for part of the year, so more goats can be grazed without decreasing cattle stocking rate. On average, breeding does weigh 150 lbs. Stocker goats are being purchased at 40 lbs. at the beginning of the time period and sold between 70 and 80 lbs. at the end of the period. At their heaviest, the stocker goats are roughly half the size of the breeding goats. Therefore, we assumed we could stock 2 stocker goats in the place of 1 breeding goat. Therefore, the stocker goat test pasture will have 16 cows and 64 goats. When breeding goats are utilized, only 2 goats will be stocked per head of cow (technology 5). Therefore, the breeding goat test pasture will have 16 cows and 32 goats. Goat operations require additional inputs such as labor, feed and medical costs. Feed requirements depend on forage availability and desired average daily gain (ADG). This study includes 2 types of feeding strategies in the stocker goat operation. In technology 3 goats are supplemented season-long to increase weight gain, while technology 4 has goats supplemented only during the late-season when forage quantity and quality is insufficient. In the breeding operation, goats will only be supplemented when necessary, to maintain a healthy weight for reproduction.

In this study, improvement in pasture productivity from woody species control is captured indirectly as reductions in supplemental feed for the cattle enterprise. It is assumed that cattle will require supplemental feed for at least some period of time in every scenario but will require less supplemental feed to maintain productivity when land is improved, resulting in a higher net present value (NPV) for the cattle operation. The cattle operation is evaluated by a stream of profits which were obtained by deconstructing the NPV values from Bir et al. [19]. This stream of profits will be incorporated into the profits and costs of the other technologies for an NPV of the entire operation. The stream of profits for the cattle operation will be input as a triangular distribution into @Risk in Excel to account for variability in forage availability [37]. @Risk triangular distribution is an excel add-on which allows for a simulation using the minimum, most likely and maximum values. Specific assumptions will be outlined in the following paragraphs.

### 2.1. Technology 1—Cattle Grazing and Traditional Woody Plant Control

Technology 1 (cattle grazing and traditional woody plant control) assumes traditional management practices utilizing the herbicide Grazon P+D which combines the 2 chemicals suggested for management [20]. Grazon P+D contains the chemicals Picloram and 2,4-D [21]. The recommended application rate for Grazon P+D is half a gallon per acre, with a cost of $121.4 per 2.5 gallons, the cost per acre comes to $24.28 [21]. Aerial spray application of the herbicide was assumed to ensure even distribution through woody areas, the cost of aerial spraying is $9.46 per acre [22]. The cost for both application and herbicide would come to $33.74 per acre or $6073.20 for the 180-acre pasture. Herbicides should be applied once every 3 years [38].

It is assumed that in technology 1, the rangeland is in poor condition (i.e., with significant woody species cover), therefore cattle will need to be supplemented additional feed in order to maintain a reasonable average daily gain. Feed costs will be assumed 50%, 40% and 30% above average when rangeland is in a poor state, therefore the annual profit assumed for the cattle portion of the operation was a minimum of $932.40, mean of $955.80 and maximum of $979.20 [19]. Again, the profit for the cattle herd (16 head) was based on the deconstruction of the NPV of a simulated cattle herd on native range with varying feed costs above and below the average base line [19].

### 2.2. Technology 2—Prescribed Fire and Cattle Grazing

Technology 2 introduces prescribed fire and eliminates the use of herbicides. The cost of prescribed fire ranges greatly depending on the amount of forage cover and the number of acres burned. In this analysis, we assume that the pastures burned are between 160 and 640 acres with midlevel eastern redcedar encroachment levels, including trees that are between 6 and 20 feet tall, and about 250 trees per acre [23]. When using hand ignition and individual tree ignition, the cost of prescribed fire would be about $18 per acre [23]. It was assumed that 1/6 of the 180-acre pasture was burned per session, and burning was performed twice a year, so each patch was burned once every 3 years. This systemic burning is often referred to as patch-burning, with species preferring to graze new growth post-burn [39]. The cost for each 30-acre burn was $540. The time period evaluated was 12 years, to allow for each patch to be burned 4 times during the study. When only prescribed fire was used, it could be assumed that feed cost for cattle would be 20%, 10% or 0% above average, resulting in a max annual profit of $1072.80, a mean annual profit of $1049.40 and a minimum annual profit of $1026.00 for the cattle portion of the operation which was input into a triangular distribution in @Risk [19,37].

### 2.3. Technology 3 and 4—Mixed Species Grazing with Prescribed Burning—Stocker Goats

When introducing mixed species grazing, there are significant additional costs. Stocker goats are used in these 2 technologies for the mixed species grazing group, meaning goats will be purchased at the beginning of each growing season and sold in the fall. It is assumed that goats will be purchased on April 1 at 40 lbs. for the market price at that time. The market price used in this analysis is sourced from the Producers Livestock Auction Company in San Angelo Texas [26]. The goats need to have supplemental feed in the winter to maintain body condition. Given the grass content in the summer, goats do not necessarily need supplementation. However, goats that are being supplemented year-round will gain weight quicker allowing for earlier sale, or sale at a heavier weight. Therefore, 2 different goat feeding strategies were evaluated. For the purpose of this study, the growing season in Oklahoma is estimated to be April through September. During this time the goats do not need to be supplemented additional feed but can be to achieve additional weight gain. Goats were purchased at 40 lbs. on April 1st in both scenarios, with an estimated average daily gain (ADG) of 0.1 lbs. if not being supplemented [27]. At the end of the growing season on 1 September, goats will need additional feed to continue gaining weight. With 0.5 lbs. of supplementation, ADG is estimated to be 0.25 lbs. per day [27]. Therefore, goats that are only supplemented from 1 September to sale can be sold at the end of November weighing 70 lbs. If goats are supplemented during the entire season, ADG is assumed to be 0.25 lbs., meaning they are sold weighing 78 lbs. at the end of September. Only 61 goats will be sold in each scenario to account for a 5% death loss.

Because they are preyed upon by many wild animals such as coyotes (*Canis latrans*), a livestock protection animal must be purchased. For this study, a livestock guardian dog was chosen, and 1 dog was placed with the herd of goats. Livestock guardian dogs typically cost $1000 and will have a working life of 6 years [36]. Therefore, this analysis assumes a dog is purchased at the beginning of year 1 and the beginning of year 7. There are additional costs associated with the use of livestock guardian dogs including dog food, routine and emergency medical costs and a small labor cost associated with the feeding and care of the dog. The annual cost of the dog is estimated to be $500 which covers potential medical costs, dog food and labor to feed and check on the dog [36]. It is assumed that dog food, medical costs and labor are divided evenly over each month, resulting in a cost of $41.67 each month.

Even though most pastures are fenced, fencing will likely need to be redone or reinforced when introducing goats. Fencing cost can vary greatly depending on the type of fence built. For this project, 4 × 4 woven wire fencing was used. The cost per foot was obtained from 3 fence companies, Twin Mountain Fence Company in San Angelo, Texas, USA [32]; the actual costs incurred in a research project at Oklahoma State University in Stillwater, Oklahoma, USA [18]; and Peck services in Canute, Oklahoma, USA [33]. The provided quotes per foot were $3.04, $3.22 and $2.75, respectively. A square 180-acre pasture requires 11,200 feet of fence. According to USDA NRCS [40], a woven wire fence should have a useful life of 20 years, therefore 60% of the cost will be accounted for over the 12 years in this study. The cost to fence the whole pasture would be $34,048, $36,064 and $30,800. These numbers were multiplied by 0.6 to account for the payback period and put into a triangular distribution in @Risk in Excel [37].

It was assumed that goats were vaccinated on arrival with CD&T. CD&T is the most common goat vaccination which provides immunity against *Clostridium perfringens* type C + D and tetanus. The CD&T vaccination should be given twice yearly. The cost of CD&T is $0.56 per head per dose or $1.12 per year. Cost was calculated based on a 100 mL bottle of CD&T from Valley Vet online with a cost of $28 assuming a 2 cc dose [34]. The goats will be treated twice annually with anthelmintic for gastrointestinal roundworms. Through analysis of a variety of deworming protocols, it is assumed that the cost of dewormer will be no more than $2 per head per dose or $4 per head per year. As some dewormers are not labeled for use in goats, an active Veterinarian-Client-Patient relationship is necessary to discuss off-label uses. The cost for vaccination and deworming per goat for 1 year comes to $5.12. Labor costs for a breeding goat operation were assumed at 3 h per goat per year [35]. The labor associated with a stocker goat operation would be significantly less and was assumed to be 1 h per year at a cost of $10 per hour. The yearly labor cost for 64 goats at 1 h of labor per goat would be $640, divided over the period the goats are grazing.

The feed utilized in this analysis consisted of 97.4% dried distiller’s grain, 1.8% calcium carbonate, 0.4% ammonium chloride and 0.4% rumensin. This ration was used in the Oklahoma State University buck test to maintain consistency with average daily gain [27]. The historical cost of dried distiller’s grain was obtained from the USDA AMS [28] website and was input into an @Risk triangular distribution to account for price changes over multiple years. The high cost of dried distiller’s grain in the ration was $253.05, the mean was $184.72 and the low was $116.39. The cost of rumensin is $656.49 for a 55 lbs. bag or $95.49 in 1 ton of the ration [29]. The cost of Calcium Carbonate is $6.85 for a 50 lbs. bag of feed or $4.93 in 1 ton of the ration, or $4.93 in 1 ton of the ration [30]. The cost of Ammonium Chloride is $7 for a 5 lb bag or $11.20 in 1 ton of the ration [31]. The percentage of each ingredient was multiplied by the cost per ton of each ingredient to obtain the cost per ton of the feed mixture. The cost of the complete ration had a high value of $364.67, a mean of $296.34 and a low value of $228.01.

When goats are added to the operation, it is assumed that the land will be improved to above average production by the reduction of woody plant encroachment. The goats are assumed to be able to reduce cattle feed costs to 20%, 30% or 40% below average, resulting in an annual profit for the cattle herd of $1143.00, $1166.40 or $1211.00.

### 2.4. Technology 5—Mixed Species Grazing with Prescribed Burning—Breeding Goats

The breeding goat operation retains many of the assumptions from the stocker goat operation, as well as introducing other variables. When using a breeding goat operation, goats will not need to be purchased each year, instead a set of does will be purchased in August of the first year and replacements will be retained each following year to account for culling or death loss. The breeding operation uses a stocking rate of 2 goats per cow, with a total of 32 does in this study. It is assumed that the does will have a weaned kid crop of 150% [41]. It is assumed that 15% of the does will be culled each year, in this operation this means 5 does will be culled each year [24]. Does will be culled in June at the same time kids are being sold. Culled does will be estimated to weigh 150 lbs and can be sold for $1.62 per lb [26]. The sale price for the culled does will be $243 per doe. The estimated death loss each year is 10% of adult does, or in this scenario 3 does [42]. This means that 8 does will have to be replaced in total as a result of culling or death loss, therefore 8 doe kids will be retained as replacements annually. When replacing 8 does a year, the original herd will be completely replaced every 4 years. A buck will also have to be purchased, it is assumed that the buck will cost $500 in year 1 and year 7 [18]. The does will be exposed to the buck on 1 October in order to kid in March. The kids can then be sold at 50 lbs in June. The cost of vaccination is $64.96 per year for the herd which includes the vaccination cost for the kids [24]. Labor requirements for a breeding operation will be higher than the labor requirements for a stocker goat operation. The labor is estimated to be an average of 3 h per doe per year spread out over each month for a cost of $80 per month [35]. Breeding goats have lower feed requirements than stocker goats because breeding goats need to achieve the appropriate weight for their stage in production rather than gain weight. The buck will be supplemented 0.5 lbs. of feed for 2 months prior to breeding to ensure adequate weight to maintain energy for breeding. The does will be supplemented 0.5 lbs. of feed during late-stage pregnancy and early lactation to meet their needs during the time of increased nutritional requirements [24]. The suckling kids will not need to receive supplemental feed because forage should be adequate for a reasonable average daily gain. The dog costs, fencing costs, fire costs and cattle value will remain the same as the stocker goat operations.

In order to determine the economic feasibility of these various types of redcedar control, an expected profit equation was determined. The expected profit equation is as follows:

(1) $$E\left[\pi_{t}\right]=\overset{N}{\underset{s=1}{\sum }}\overset{A}{\underset{k=1}{\sum }}\overset{F}{\underset{d=1}{\sum }}\overset{E}{\underset{b=1}{\sum }}.\overset{R}{\underset{m=1}{\sum }}\overset{L}{\underset{c=1}{\sum }}(Revenue_{dskm}-Cost_{sdbm})+Profit_{cm}|t$$

where *E*[π*_t_*] is the expected profit, s is the number of stocker goats on the operation from 1 to *N*, *k* is the number of kids in the operation from 1 to *A*, *d* is the number of does from 1 to *F*, *b* is the number of bucks from 1 to *E*, m is month from 1 to *R*, *C* is the number of cattle from 1 to *L* and t denotes the technology used. Where *t* = 1 is standard control methods, *t* = 2 is an operation utilizing prescribed fires, *t* = 3 is an operation using goats supplemented season-long and prescribed fires as the control method, *t* = 4 is an operation using goats supplemented only in the late-season and prescribed fires as the control method and *t* = 5 is the breeding goat operation. The revenue equation includes potential income related to any of the 5 technologies. This equation is as follows:

(2) $$Revenue_{t}=\overset{N}{\underset{g=1}{\sum }}.\overset{R}{\underset{m=1}{\sum }}.(W_{skm}\times PG_{m})+243Does|t$$

where *W* is the weight of goats at sale either kids or stocker goats depending on the technology, *PG* is the price of goats when sold. Does is the number of does sold multiplied by $243 which is the approximate sale price of mature does at auction. The equation for the number of kids sold for technology 5 is as follows:

(3) $$KidsSold=\overset{A}{\underset{k=1}{\sum }}\left(Does\times 1.5\right)-8$$

The breeding goats have a weaned kid rate of 150% which corresponds to the 1.5 in Equation (3). Here, 8 doe kids are retained each year as replacements for does who died or were culled, so 8 less kids are sold each year than are born. The equation for the weight of goats at sale when fed only in the late-season is given below:

(4) $$Weight=\overset{150}{\underset{day=1}{\sum }}ADG_{1}\times 150+\overset{210}{\underset{day=150}{\sum }}ADG_{2}\times 60$$

Equation (4) will only be used in technology 4 when the kids are supplemented feed outside of the growing season. In Equation (4), *ADG*1 is 0.1 lbs. per day when not supplemented feed and *ADG*2 is 0.25 when supplemented feed. The kids born from the breeding operation are not supplemented feed and can be assumed to gain 0.1 lbs. per day as corresponds to *ADG*1. The equation for weight of goats at sale when supplemented feed all year is given as:

(5) $$Weight=\overset{180}{\underset{day=1}{\sum }}ADG_{2}\times 150$$

The cost equation consists of all potential costs related to the use of technology 1, 2, 3, 4 or 5. This equation is given as:

(6) $$\begin{array}{ll} Cost_{t}=\overset{N}{\underset{g=1}{\sum }}.\overset{R}{\underset{m=1}{\sum }}. & \left(IG_{sm}\right)+\left(IG_{bm}\right)+\left(IG_{dm}\right)+\left(ID_{m}\right)+\left(FC_{m}\right)+\left(F_{m}\times A_{m}\right)+\left(MED_{sbdm}\right)\\ & +\left(LG_{dsm}\right))+\left(SUPP_{dbsm}\right)+\left(DF_{m}\right)+\left(CHEM_{m}\right)|t \end{array}$$

where *IG* is the initial purchase of the goats, *ID* is the initial purchase of the livestock guardian dogs, *FC* is the cost to build fence and *F* is the cost of prescribed fire per acre times the amount of acres burned. *MED* signifies the cost of providing routine and emergency medical care to the goats. *LG* denotes the labor costs related to goats. *SUPP* is the additional costs of feed and free choice minerals for the goats and *DF* is the cost of dog food and other additional costs related to the livestock guardian dogs. *CHEM* is the cost of herbicide use and application.

## 3. Results

Table 2 presents the results for the NPV of each of the five technologies: traditional management, prescribed fire, a stocker goat operation with feed supplemented in late-season only, a stocker goat operation with feed supplemented season-long and a breeding goat operation. Technology 1, the use of standard control using herbicide has a mean NPV of −$11,068.85. This analysis showed that there is a 0% probability of a positive NPV with standard control methods. The use of fire as a control method has a NPV of −$318.12, with a 0% probability of a positive NPV given the assumptions of this analysis. Both the third and fourth technologies when stocker goats are introduced have negative mean NPVs with a 0% probability of a positive NPV. When feeding the goats season-long, the NPV is −$19,893.61, and when feeding goats in the late-season only the NPV is −$21,259.65. This shows that supplementing the goats only in the late-season is slightly more profitable than feeding season-long. When a goat breeding operation is utilized, a positive NPV results. The mean NPV of the breeding operation is $5503.09, which is the highest mean NPV of the five technologies. The simulation showed that the breeding operation has a 99.9% probability of a positive NPV. In this analysis the breeding operation has the highest profitability and is the only profitable choice based on these assumptions and ideal practices.

## 4. Discussion

The results show that the breeding operation is the only economically feasible control method for woody plant control in this analysis. The use of the goat breeding operation in conjunction with cattle production is likely the most economically feasible as well as the most effective for woody plant control. The use of prescribed fire is close to a positive NPV in the simulation, and given different assumptions there is a possibility for a positive NPV. As additional real-world research is conducted, more specific numbers can be used in the simulations of the technologies studied. Traditional control appears to not be profitable, and also likely does not control eastern redcedar encroachment nearly as well as the other methods. It is important to note that many assumptions were made regarding the use of best-practices. It is possible that many producers are not using herbicides at the recommended rate or amount. Even though this would result in decreased costs, this would also result in decreased control.

**Figure 1:**
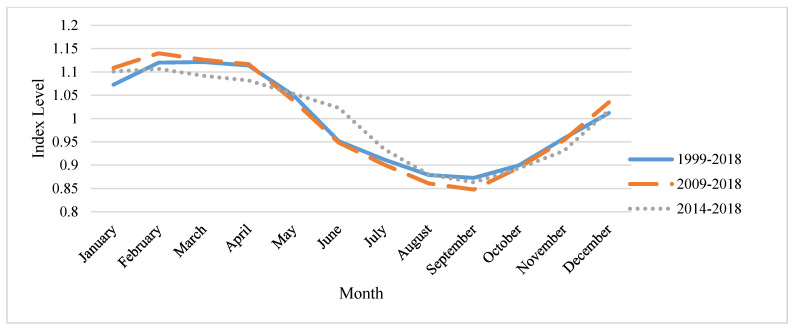
Monthly producer price index for the sale of 60 to 80 lb goat kids.

One factor which may have played a role in making the stocker goat operation not profitable was the seasonal price differences. A graph of the seasonal price index is shown in Figure 1. This figure shows the price indices for three time periods: 1999–2018, 2009–2018 and 2014–2018. In the final time period, it appears that the price may be leveling out and over time, showing that seasonal price differences may be insignificant. In the stocker goat operation, the goats are purchased in April, which is one of the months with the highest prices for goats. The seasonal price index value for April is 1.1136, meaning that prices in April are 11.36% higher in April than the average throughout the whole year. Not only were the goats purchased at a high price, but they were sold in September and November when goat prices are at a low point for the year. We chose to utilize stocker goats during this time because this is the main growing season for Oklahoma therefore the goats will have the greatest amount of available forage. In September, the seasonal price index is 0.8726 and in November the seasonal price index is 0.9574, meaning that the prices are 12.74% and 4.26% lower than the yearly average, respectively. A benefit of the use of a breeding operation is that a producer can choose when to breed and sell the goats to meet the demand in the market when prices are high given biological constraints.

The cost to build a fence is significant in all of the technologies related to goats. The fence chosen in the study is 4 × 4 net wire which is the best and most expensive fence available. This fence will be the most effective at keeping goats in and predators out, but there are other less expensive options which could be utilized. If a producer already has adequate fencing, the breeding goat operation would have a much higher NPV and the stocker operation may be brought to a positive NPV. The cost of fence can be reduced by improving existing fence if insufficient fence is already present [18]. If a producer already has a five-strand barbed wire fence in good condition, the fence could be improved in multiple ways. The most cost-effective method to improve this fence would be adding two additional strands of fence, these additional strands would be electric. The cost of the wire, fence charger, insulators and labor for these improvements would be about $1978.75. The addition of three strands of electric fence would cost about $2295.77. Additional strands of barbed wire could also be added to improve fence. The cost for three additional barbed wires is $2760.10 and the cost for five additional strands is $4043.50. The cost includes the wire, t-post clips and labor to improve the fence and clear the fence line. It is clear that this cost to improve the fence is significantly lower than the cost to build a new fence with 4 × 4 mesh wire, however there are potential issues to consider when choosing the type of fence. The 4 × 4 mesh wire fence will keep goats in and predators out better. If barbed wire or hot wire fence is chosen, a producer should expect to have a larger percentage of goats lost to predators or escape than if 4 × 4 net wire is used.

Another impactful cost of the stocker goat operation was the purchase of goats every year. When the producer uses a breeding operation, it will negate some of the costs associated with a stocker goat operation. A breeding goat operation introduces additional costs such as the maintenance of a buck and additional labor, but these costs are less than the costs of purchasing stocker goats each year. In addition to all the cost variables associated with these technologies, a conservative method for estimation of land value was used and the goats and prescribed fire may improve the land more than estimated. Stocking rates can be highly variable when considering forage quality. It is estimated that the optimal stocking rate and profitability decrease in situations with increased precipitation variability due to climate change [43]. Even though stocking rate was held constant in this analysis, depending on the level of forage improvement, evaluation of stocking rate may be another way to evaluate differences between these scenarios.

According to a study on meat goat farm efficiency, farm size has a significant impact on profitability of a meat goat operation [24]. It appears that as farm size grows, the operation becomes more profitable. Therefore, if this study was performed on a pasture larger than 180 acres, and stocked with more goats, profitability would likely increase. According to Qushim et al. [24] a goat operation must have greater than 40 breeding does to be profitable. Our study found that even with 32 breeding does the operation was profitable alongside a cow-calf operation, but this shows that by increasing the number of does, profitability could increase.

A conservative estimate of potential woody plant control was assumed in this analysis. It is possible the level of land improvement may vary depending on the level of woody plant encroachment and the individual grazing pattern of the goats. Previous work in sheep found that rams’ ability to detect the bitter-tasting compounds that discourage other animals like cattle from eating shrub encroachment varies between individuals [44]. This indicates that it may be possible to selectively breed for goats or sheep that have a stronger preference for these bitter tasting plants. Even though this will not affect profitability of the goat herd itself, given our assumptions of selling at a set weight, it may help improve the land, through shrub reduction at a faster rate.

According to Stritzke and Bidwell [45], most traditional chemicals used for weed control is not effective on eastern redcedar trees, so while the use of chemical control appears to be less expensive than the use of mixed species grazing, it may not be as effective. Not only are there concerns with the effectiveness of herbicides, but increased regulations may make the use of herbicides for woody plant control not feasible. Because herbicides are often overused in the management of crop land, herbicide resistant weeds have evolved [46]. A solution to this is implementing changes in herbicide use through regulations [46]. The use of mixed species grazing and prescribed fires both appear to be more effective control methods than the use of herbicides. Future studies may be able to evaluate the effectiveness and profitability of the use of these methods through real world analyses of the change in average daily gain of cattle or increased stocking rate. Previous studies have found that some segments of cattle producers are willing to reduce stocking rates to achieve grassland conservation outcomes, for invasive cool-season grass that favors heavy grazing [47]. Even though we assumed appropriate stocking rates based on the literature, many producers are overstocking cattle. Improving forage quality may simply bring overstocking producers to appropriate stocking levels without the economic hit of decreasing herd size. One aspect that may impact whether cattle producers decide to include goats as part of their rangeland management strategy is social norm pressures. Studying the management of non-native grass through practices such as herbicide application, prescribed fire and physical removal, Coon et al. (2020) found that both personal and social norms along with attitude impacted willingness to control non-native grasses [48].

## 5. Conclusions

This study found that the most economically feasible, and effective control methods would be the utilization of a mixed species grazing operation utilizing breeding goats, combined with prescribed burning. Prescribed fire has a negative NPV in the simulation but is close enough to a positive NPV that with some cost cutting measures or different assumptions, the NPV could become positive. Traditional management appears to be both expensive and not effective. The difference in average simulated NPV between the traditional management method and the breeding goat enterprise was approximately $16,500. Both stocker goat operations, goats supplemented season-long and goats only supplemented late-season have significantly negative NPVs and appear to not be economically feasible. While neither of these methods should be chosen in the presence of other options, feeding stocker goats late-season only appears to be a better method of supplementation. Because of the great number of variables and differences in production, additional studies should be conducted to obtain an accurate understanding of the methods of woody plant control. A real-world analysis would offer a more complete understanding of the economic feasibility of mixed species grazing, pyric herbivory and patch burning.

## Figures and Tables

**Table 1:** Assumptions for the cost benefit analysis of the 5 technologies. (1) The standard woody plant control method with cattle grazing, (2) use of prescribed fire with cattle grazing, (3) use of fire, stocker goats and cattle grazing with goat feed supplementation during the entire season, (4) use of fire, stocker goats and cattle grazing with goat feed supplementation in the late season only and (5) the use of fire, breeding goats and cattle grazing with goats only supplemented when necessary for breeding.

Item	Technology 1	Technology 2	Technology 3	Technology 4	Technology 5	Source
No. of cows	16	16	16	16	16	[18]
No. of acres	180	180	180	180	180	-
Cattle Value	150%, 140%, 130% feed cost	120%, 110%, 100% feed cost	60%, 70%, 80% feed cost	60%, 70%, 80% feed cost	60%, 70%, 80% feed cost	[19]
Herbicide Cost	$6073.20 every 3 years	-	-	-	-	[20,21,22]
Fire Cost	-	$540 twice a year	$540 twice a year	$540 twice a year	$540 twice a year	[23]
No. of Goats	-	-	64; 5% loss; 61 sold	64; 5% loss; 61 sold	32	[24]
Goat Purchase Date	-	-	April 1 at 40 lbs	April 1 at 40 lbs	Does: 1 August of year 1 at 70 lbs Buck: $500 Year 1 and Year 7	-
Goat Purchase Price	-	-	Low: $1.57 Mean: $2.44 High: $3.15	Low: $1.57 Mean: $2.44 High: $3.15	Low: $1.23 Mean: $2.16 High: $2.40	[25]
Goat Sale Date	-	-	1 September at 78 lbs	1 November at 70 lbs	40 kids sold per year in June at 50 lbs	-
Goat Sale Price	-	-	Low: $1.22 Mean: $1.73 High: $2.38	Low: $1.22 Mean: $1.99 High: $2.47	Low: $1.295 Medium: $2.27 High: $265.16	[26]
Average Daily Gain	-	-	0.25 lbs	0.1 lbs 1 April–1 August; 0.25 lbs August 1– November 1	-	[27]
Days Fed (stocker goats)	-	-	1 April–1 September (150 days fed; 0.5 lbs/head)	1 August–1 November (60 days fed; 0.5 lbs/head) (150 days not fed)	-	-
Feed Cost (per ton)	-	-	Low: $228.01 Mean: $296.34 High: $364.67	Low: $228.01 Mean: $296.34 High: $364.67	Low: $228.01 Mean: $296.34 High: $364.67	[27,28,29,30,31]
Goat Fence Cost	-	-	Low: $18,480.00 Mean: $20,428.80 High: $21,638.40	Low: $18,480.00 Mean: $20,428.80 High: $21,638.40	Low: $18,480.00 Mean: $20,428.80 High: $21,638.40	[18,32,33]
Goat Vaccination	-	-	$5.12/year/goat	$5.12/year/goat	$64.96 covers kids and does (per year for the herd)	Stockers: [24]; Does: [34]
Goat Labor	-		$106.67/month when goats present	$80/month when goats present	$80 per month	[35]
Buck Feed	-	-	-	-	August and September only; 0.5 lbs/head	[24]
Doe Feed	-	-	-	-	January through April only; 0.5 lbs/head	[24]
Initial Cost of Dog	-	-	$1000 in years 1 and 7	$1000 in years 1 and 7	$1000 in years 1 and 7	[36]
Dog Costs	-	-	$41.67 per month	$41.67 per month	$41.67 per month	[36]

**Table 2:** Table of simulated NPV results for the four management options studied.

Management Type	Mean NPV	Standard Deviation of NPV	Minimum NPV	Maximum NPV	Probability of a Positive NPV
Traditional Management	−11,068.85	24.79	−11,144.73	−11,00.97	0%
Prescribed Fire	−318.12	24.41	−397.79	−241.64	0%
Goats-Fed Season-Long	−19893.61	3805.10	−32695.38	−6188.85	0%
Goats-Fed Late-Season	−21259.65	3801.36	−32641.19	−9513.76	0%
Breeding Operation	5503.09	1738.64	−195.68	10977.81	99.9%

## Data Availability

All data sources are disclosed in the document and publicly available.
